# Workplace Bullying and Suicidal Ideation: Findings from an Australian Longitudinal Cohort Study of Mid-Aged Workers

**DOI:** 10.3390/ijerph17041448

**Published:** 2020-02-24

**Authors:** Liana S. Leach, Lay San Too, Philip J. Batterham, Kim M. Kiely, Helen Christensen, Peter Butterworth

**Affiliations:** 1 National Centre for Epidemiology and Population Health, Research School of Population Health, The Australia National University, Canberra, ACT 0200, Australia; 2 Centre for Mental Health, Melbourne School of Population and Global Health, The University of Melbourne, Parkville, VIC 3052, Australia; tiffany.too@unimelb.edu.au; 3 Centre for Mental Health Research, Research School of Population Health, The Australia National University, Canberra, ACT 0200, Australia; Philip.Batterham@anu.edu.au; 4 School of Psychology, Faculty of Science, University of New South Wales, Sydney, NSW 2052, Australia; k.kiely@unsw.edu.au; 5 Neuroscience Research Australia (NeuRA), Sydney, NSW 2031, Australia; 6 Black Dog Institute, University of New South Wales, Sydney, NSW 2052, Australia; h.christensen@blackdog.org.au; 7 Melbourne Institute of Applied Economic and Social Research, The University of Melbourne, Parkville, VIC 3052, Australia; peter.butterworth@anu.edu.au; 8 Centre for Research on Ageing, Health & Wellbeing, Research School of Population Health, The Australia National University, Canberra, ACT 0200, Australia

**Keywords:** workplace bullying, mobbing, suicide, suicidal ideation

## Abstract

Workplace bullying adversely affects mental health, yet little is known about the outcomes for suicidal ideation. The current study used Australian population-based data to investigate the association between workplace bullying and suicidal ideation. The sample included 1488 employed participants aged 52–58 from wave 4 of the Personality and Total Health (PATH) Through Life Study. Workplace bullying was measured in two ways: (a) a single item asked about experiences of bullying ‘currently’, ‘previously in the current workplace’ and ‘in a past workplace’, and (b) 15 items asked about bullying behaviours experienced in the past 6 months. Suicidal ideation was measured using items from the Psychiatric Symptom Frequency Scale (PSF) and the Patient Health Questionnaire-9 (PHQ-9). Psychosocial job quality, both current and prior, was adjusted for. Current and past experiences of workplace bullying were associated with increased risk of suicidal ideation. Current experiences were no longer associated after adjusting for concurrent indicators of psychosocial job stress, although a tendency for increased ideation remained. Reported prior experience of workplace bullying in a *past workplace* remained associated with higher odds of suicidal ideation after adjusting for prior psychosocial job stressors and excluding individuals with prior suicidal ideation. Being bullied at work is associated with increased risk of suicidal thoughts, although this occurs within the broader influence of other psychologically stressful employment conditions.

## 1. Introduction

Workplace bullying is a serious problem occurring in a variety of workplaces globally. Workplace bullying or ‘mobbing’ refers to interactions in the workplace where a person is the target of repeated and persistent negative behaviour and/or abuse from others within the organisation. It is typically accompanied by a power imbalance (either structural or social) between the person/s performing the behaviour and the person being targeted [1]. Research suggests workplace bullying is common; a 2010 meta-analysis reported 14.6% point prevalence (CI: 12.3% to 17.2%; including 70 studies—most of which reported a 6–12 month prevalence) [2]. In addition to being common, workplace bullying has adverse outcomes for both the workplace and the individual. The costs to the workplace (and employers) include higher rates of absenteeism, presenteeism and staff turnover. Individuals who experience bullying take more sick leave [3] and report decreased job commitment and satisfaction [4]. There is also clear evidence that workplace bullying is associated with increased mental health problems for victims, from meta-analyses and systematic reviews [2,5,6]. 

In contrast to the substantial evidence base linking workplace bullying with common mental health problems, such as depression and anxiety, far less research has explored the potential for increased suicidal ideation and behaviour. Suicide is a major public health concern worldwide, with approximately 800,000 deaths by suicide every year [7]. The World Health Organisation (WHO) recognises suicide as a public health priority. The WHO member states are working towards a 10% reduction in global suicide by 2020 [7]. While there is widespread concern about suicide, research efforts to identify new, emerging risk factors for suicidality (and targets for prevention) have been sporadic. For example, a recent meta-analysis [8] noted that most population-based research has been restricted to the roles of pre-existing mental illness, previous suicidality and demographic factors. While there has been new research on the role of interpersonal factors, social isolation, and school bullying [9,10], few studies have focused on the work environment (where many adults spend most of their time) as a source of interpersonal stress.

Research that has explored workplace bullying and suicidal ideation does support an association. However, there are major gaps in the existing literature. A recent systematic review [11] identified two relevant longitudinal, population-based studies and eight cross-sectional studies [12,13]. Both longitudinal studies utilised data from the same national register of employees in Norway, with participants followed up two and five years after workplace bullying was experienced. The first study found that those who reported bullying had twice the odds of suicidal ideation at a later time point [13]. The second study indicated that only behaviour involving physical intimidation predicted suicidal ideation two and five years later [12]. However, neither of these longitudinal studies controlled for other work-related adverse psychosocial risk factors, such as job insecurity, low job control, or high job demands/intensity. In fact, regardless of whether existing relevant studies are cross-sectional or longitudinal, the systematic review noted that far more research is needed to discern the extent to which bullying contributes to suicidal ideation independent of other psychosocial work-related factors, given that there is evidence of an association between psychosocial working conditions and suicidal ideation [14,15]. 

The systematic review [11] also highlighted a clustering of research evidence available in European countries, and very little elsewhere. Of the 12 studies reviewed, only two were conducted outside Europe—one in Canada and the other in Australia. The Canadian study was cross-sectional (n = 1082) [16] and found that those who were bullied at work (currently or in the past year) had greater suicidal thoughts; however, no socio-demographic factors nor workplace characteristics were controlled for. The Australian study was cross-sectional and nationally representative (n = 932) [14]. The analysis adjusted for demographic factors and was the only published identified to have additionally adjusted for work-related factors (i.e., supervisor support, job control, demands and insecurity). The results showed that workplace bullying was independently associated with twice the odds of suicidal ideation. However, one limitation is that the measure of suicidal ideation was not specifically designed to assess suicide—it was taken from the broader Beck Depression Inventory and did not discriminate between active and passive suicidal thoughts (indicating a need to further replicate these results).

Overall, methodologically robust population-based studies investigating the association between workplace bullying and suicidal ideation remain rare. The current study is one of the first to investigate this association while adjusting for other established psychosocial job stressors (i.e., job demands, control and insecurity) and only the second population-based study to be conducted with an Australian community-based sample. While the study essentially uses cross-sectional data, we adopt a measure of workplace bullying that asks about both current and past experiences (i.e., a self-labelling approach), as well as a measure that explores the potential impacts of person-related, work-related and physically-related bulling behaviours (i.e., a behaviour-based approach). Improving on past research, our measure of suicidal ideation is well validated and targeted at active (rather than passive) suicidal ideation—as active ideation is more closely correlated with suicidal behaviour. In addition, we exclude those who report past suicidal ideation (at a prior wave of data collection four years earlier) to more confidently assume onset. 

## 2. Materials and Methods

### 2.1. Participants

Participants were from the Personality and Total Health (PATH) Through Life Study undertaken by the Centre for Research on Ageing, Health and Wellbeing at The Australian National University [17]. PATH is a longitudinal community survey about the health and wellbeing of young (20–24), midlife (40–44), and older (60–64) adults. Participants were randomly selected from the Australian Electoral Rolls of Australian Capital Territory and the neighbouring town of Queanbeyan. They were first assessed in the year 2000 and have been followed up every four years. 

While the PATH study is longitudinal, this study focuses on cross-sectional data collected from the mid-aged cohort at wave 4 (in 2012, aged 52–58 years), as this is the only wave when measures of workplace bullying were included. At this time, 2257 participants remained in scope (of the original 2404 interviewed at wave 1) and were invited to complete the wave 4 online survey. Overall, 1806 participants (80%) completed the survey. Participants were excluded from the analysis if they were unemployed, not in the labour force, employed but on long-term leave, or had missing data on employment status (n = 340) or if they did not complete the items on workplace bullying (n = 18). The final analysis sample included 1488 participants. The PATH project has been approved by the Human Research Ethics Committee of the Australian National University (approval numbers M9807, 2002/189, 2006/314, 2010/542, 2016/445) and all participants provided written informed consent.

### 2.2. Exposure Measures

Self-labelling and behaviour-based questions were used to assess workplace bullying at wave 4. The self-labelling question asked ‘*Mental violence or workplace bullying refers to isolation of a team member, underestimation of work performance, threatening, talking behind one’s back or other pressurising. Have you experienced such bullying?*’ Participants responded either ‘Never’, ‘Yes, currently’, ‘Yes, previously in this workplace’, ‘Yes, previously in another workplace’, or ‘Cannot say’. This self-labelling approach has been used previously to estimate prevalence of workplace bullying [18,19]. The behaviour-based question asked *‘How often have any of the following occurred to you in your workplace over the past 6 months? Choose the response closest to your experiences’*. Fifteen items were presented, such as ‘Being ignored or excluded’ (see prior studies for a full list [20,21]), and participants were asked to indicate either ‘Never’ (1), ‘A few times’ (2), ‘Sometimes’ (3) or ‘Often’ (4). Previous factor analysis of these items [22] suggests a three-factor solution best represents the data, and differentiates between person-related bullying, work-related bullying, and violence and intimidation. Thus, three corresponding scales ranging from 1 to 4 were calculated by averaging the scores of all relevant items.

### 2.3. Outcome Measure

Suicidal ideation was assessed using five items at wave 4: four from the Psychiatric Symptom Frequency Scale (PSF) [23] and one from the Patient Health Questionnaire-9 (PHQ-9) [24]. The four PSF items were: *‘In the last year have you ever felt that life is hardly worth living?’*; *‘In the last year have you ever thought that you really would be better off dead?*’; *‘In the last year have you ever thought about taking your own life?’; and ‘In the last year have you ever thought that taking your life was the only way out of your problems?’*. The first two items capture passive ideation (or a passive wish to die), whereas the final two capture active ideation, with the implication being that passive ideation does not have the same life-threatening context [25,26]. Participants were instructed to indicate ‘Yes’ or ‘No’. The item from the PHQ-9 was *‘Over the last 2 weeks, how often have you thought that you would be better off dead or of hurting yourself in some way?’*, rated ‘Not at all’, ‘Several days’, ‘More than half the days’, or ‘Nearly every day’. The outcome for the main analyses was ‘active’ suicidal ideation based on a response of ‘Yes’ to either of the final two PSF items, or at least ‘Several days’ to the PHQ-9 item. Sensitivity analyses adopted a broader definition of ideation that included a positive response to any of the PSF/PHQ-9 items. Measures of prior suicidal ideation were similarly derived from the wave 3 survey data (four years earlier).

### 2.4. Covariates

Covariates included socio-demographic variables (sex, age, partner status, education, household income, employment status, employment sector, occupational skill level, and working hours) and number of chronic physical health conditions (i.e., epilepsy, asthma, bronchitis, emphysema, diabetes, thyroid problems, arthritis, Parkinson’s disease, heart problems, stroke, transient ischaemic attack or cancer). Details of the categories for each covariate are in Table 1. Psychosocial job adversities (both current (i.e., wave 4) and at the prior wave four years earlier (i.e., wave 3)) were also considered, including low job control, high job demands, and job insecurity. Job demands and job control were assessed using 19 items from the Whitehall II study [27], which comprised four items on job demands and 15 items on job control. Scales representing job demands and job control were calculated by summing all relevant items and were then dichotomised at the tertile corresponding with greatest adversity (i.e., high job demands, low job control) [28,29]. Perceived job insecurity was measured by the item *‘How secure do you feel about your job or career future in your current workplace?’* Possible responses were ‘not at all secure’, ‘moderately secure’, ‘secure’ and ‘extremely secure’. Individuals who selected either ‘not at all’ or ‘moderately secure’ were categorised as having job insecurity. 

### 2.5. Statistical Analyses

Descriptive statistics described the sample and experiences of workplace bullying, both for the whole sample and for those with and without active suicidal ideation. Logistic regression models assessed the association between workplace bullying and active suicidal ideation. These included the simple association (Model 1), adjusting for socio-demographic covariates (Model 2), and adjusting for psychosocial job characteristics (Model 3). Thus, in Model 3, both current and prior (wave 3) measures of psychosocial job characteristics were included to account for their impact on both current and prior experiences of workplace bullying. A final model (Model 4) excluded respondents with active suicidal ideation at the prior wave (approximating the onset of suicidal ideation since the last measurement occasion). Subsequent supplementary analyses considered the additional effect of depression [from the PHQ, [24]] and neuroticism [from the EPQ, [30]]. The main models were repeated for each of the behaviour-based measures of bullying, with each of the continuous scales recoded such that a one unit difference represented the difference between the 25th and 75th percentile. Cases with missing data were minimal (96.5% had no missing data on any variables in the final model) and we report complete-case analysis.

## 3. Results

### 3.1. Descriptive Information

Active suicidal ideation in the prior 12 months was reported by 8.4% of respondents (13.4% reported either active or passive ideation). A current experience of workplace bullying was reported by 7.0% of respondents, with a further 16.6% reporting a previous experience in their current workplace, and 22.8% in a previous workplace. Table 1 shows the characteristics of the sample. In the overall sample, the average age was 55 years, just over half respondents were female, the majority was married or in a marriage-like relationship, and just over half worked in the public sector (either for the Commonwealth or Territory/State Governments). The reported household income of respondents (median $1700–$2400 per week) was consistent with the national reported median income for adults aged 45 to 54 years, $1927 [31]. Those with active suicidal ideation in the past 12 months were significantly more likely to have a lower weekly household income, to have low control and low security in their jobs, and to report experiences of current or past workplace bullying. Those with active suicidal ideation also had more experiences of person-related and work-related bullying in the past 6 months, compared to those without active suicidal ideation.

### 3.2. Workplace Bullying (Current and Past) and Suicidal Ideation

Table 2 shows the results from logistic regression models examining the association between workplace bullying (using the self-labelling measure) and active suicidal ideation. Model 1 shows that compared with no experience of workplace bullying, current bullying was associated with over two and a half times the odds of suicidal ideation. Prior experiences of bullying in the current workplace or in a previous workplace were also associated with twice the odds of suicidal ideation. These results were minimally changed by the inclusion of socio-demographic and health covariates (model 2), and the associations remained statistically significant. 

In model 3, current and prior (four years earlier) measures of psychosocial job adversity were added. Preliminary analyses explored the association between each of the psychosocial job adversity measures and workplace bullying. There was a significant positive association between workplace bullying and each of the job adversities (job control: χ^2^ (*df* = 4) = 41.4, *p* < 0.001; job demands: χ^2^ (4) = 14.3, *p* = 0.006; job insecurity: χ^2^ (4) = 38.8, *p* < 0.001) and with a measure representing any experience of these adversities (χ^2^ (4) = 29.0, *p* < 0.001). Overall, 83% of those reporting current workplace bullying also reported one of these adversities. In contrast, 58% of those who reported no workplace bullying also reported one of these adversities. When psychosocial workplace adversities were included in model 3, this substantially reduced the association between current workplace bullying and suicidal ideation, such that it was no longer statistically significant. While prior bullying in the same workplace and in a previous workplace remained significantly associated with suicidal ideation, all three bullied groups were similarly about twice as likely to experience suicidal ideation compared to those who had never been bullied. A final model (model 4) was restricted to respondents who did not report active suicidal ideation at the previous wave (n =1301). In this final model, while both current and past experiences of bullying continued to show an elevated likelihood of suicidal ideation, only bullying in a previous workplace was significantly associated with current ideation. 

Supplementary analyses additionally included (further to model 4) a measure of past depression and a measure of neuroticism to account for their potential association with active suicidal ideation. In the first analyses including prior depression, the association between bullying in a previous workplace and suicidal ideation remained statistically significant (OR = 2.21, 95% CI = 1.11 to 4.40). Current and past bullying in a present workplace were not significantly associated, although the odds remained elevated (OR = 1.49, 95% CI = 0.50 to 4.45; OR = 1.52, 95%CI = 0.70 to 3.30). In the second analyses including a measure of neuroticism, none of the workplace bullying groups had significantly elevated odds of active suicidal ideation (OR = 1.54, 95% CI = 0.50 to 4.72; OR = 1.39, 95% CI = 0.63 to 3.06; OR = 1.89, 95% CI = 0.93 to 3.83). Additional sensitivity analyses repeated the main analyses but adopted a broader measure of suicidal ideation including both passive and active ideation (see Supplementary Table S1). Differences were that in Model 3, when psychosocial job adversities were adjusted for, current workplace bullying remained significantly associated with the broader measure of ideation (likely reflects the higher prevalence of passive ideation). In Model 4, only bullying in a previous workplace remained significantly associated with ideation after excluding those with prior suicidal ideation. This remained the case after adjusting for prior depression (OR = 2.44, 95% CI = 1.45 to 4.11) and neuroticism (OR = 2.22, 95% CI = 1.30 to 3.78).

### 3.3. Person-, Work- and Physically-Related Bullying Behaviours and Suicidal Ideation

Simple unadjusted analysis of the behavioural dimensions of current workplace bullying showed that person-related and work-related behaviours were associated with increased odds of active suicidal ideation (OR = 1.42, 95% CI = 1.16 to 1.73; OR = 1.74, 95% CI = 1.36 to 2.22). The unadjusted model examining the measure of violence and intimidation showed an association in the same direction, but this was not statistically significant (OR = 1.39, 95% CI = 0.72 to 2.70). After controlling for other psychosocial workplace adversities (both current and prior), person-related bullying remained significantly associated with active suicidal ideation (OR = 1.41, 95% CI = 1.02 to 1.97), although work-related experiences did not (OR = 1.13, 95% CI = 0.83 to 1.54). After excluding those with prior suicidal ideation four years earlier, none of the workplace bullying behaviour categories were significantly associated with active ideation.

## 4. Discussion

The results suggest that those who are currently or who have previously experienced workplace bullying are at greater risk of suicidal ideation. There was some attenuation of the odds ratios and statistical significance when covariates (particularly psychosocial job adversities and neuroticism) were adjusted for, pointing towards the interplay with these factors (as well as the low prevalence of suicidal ideation. Nevertheless, workers who reported an experience of bullying had consistently 1.5–2 times higher odds of active suicidal ideation, suggesting that workplace bullying maintains an independent influence. Overall, the findings raise important questions about the timing and severity of workplace bullying experiences, the conceptual overlap and/or co-occurrence of bullying with other aspects of psychosocial job adversity, and how these factors might influence suicidal ideation.

In the current study, we see that workplace bullying seems more likely to occur within the context of poorer psychosocial job quality, particularly low control and insecurity. It is not surprising that a range of adverse characteristics, including workplace bullying, cluster together within poor quality jobs or that the independent influence of current bullying was reduced after accounting for job demands, control and insecurity (i.e., from odds of 2.37 to 1.98). This was similar in the analyses using the behaviour-based measures of work-related bullying (although it should be noted that the measure of workplace bullying included items related to job strain and insecurity). This aligns with research showing that the association between workplace bullying and depression/anxiety attenuates when psychosocial employment conditions are accounted for [21,32]. While this could indicate conceptual overlap between workplace bullying and psychosocial adversity, our prior investigations suggest that while correlated, they are reasonably independent [21]. In part, our findings contradict the only other Australian study to investigate workplace bullying and suicidal ideation (and as far as we know the only other study internationally to adjust for psychosocial job stressors). Milner et al. found that the association remained statistically significant after including psychosocial job stressors [14]. However, Milner et al. also found substantial change in the odds ratios before and after adjustment, consistent with the current study (i.e., unadjusted OR = 2.09, 95% CI = 1.67–2.64; adjusted OR = 1.54, 95% CI = 1.16–2.05).

The enduring association between bullying in a prior workplace and active suicidal ideation is important to consider. This result suggests the effect of workplace bullying are persistent and not restricted to the time or place it occurs. This finding echoes studies by Nielsen et al. [12,33], showing that increased suicidal ideation is maintained two and five years after bullying. Verkuil and colleagues also propose that the mental health impacts continue over time, as invasive experiences are recreated in intrusive thoughts, rumination and prolonged stress [6]. One further possibility in the current study is that individuals who reported bullying in a prior workplace recalled particularly toxic/destructive experiences and left these workplaces as a result. This possibility accords with previous research showing that severity of workplace bullying is associated with greater turnover intentions and actions [34]. While the current study raises these possibilities, the lack of prospective longitudinal data assessing workplace bullying, and the imperfect adjustment for prior psychosocial job adversity (i.e., it may not concord with the timing of prior bullying) limits our ability to contrast the effects of current and past experiences. Overall, however, we conclude that both are likely important. In addition, further in-depth qualitative research specifically exploring the course of impacts (over the short to long-term) would assist in our understanding.

In terms of future research directions, there is much potential to better identify the causal and temporal links between workplace bullying and suicidal ideation using prospective longitudinal data with multiple time points. The current study suggests these data should include robust, time-based measures of workplace bullying and suicidal ideation, structural and psychosocial job characteristics, job turnover, and neuroticism. There is also the potential to include measures that assess the social processes involved in workplace bullying to link in with emerging interpersonal theories of suicidality. For example, the Interpersonal-Psychological Theory of Suicide (IPTS) [35] purports that the key components necessary for suicidal thoughts are ‘thwarted belongingness’ (social isolation from a valued social circle) and ‘perceived burdensomeness’ (the perception of being a burden on others with little hope of change). If we apply the IPTS model to the context of workplace bullying, there are clear synergies with feelings of social isolation and an inability to escape the situation [33,36]. 

### Limitations

This study has several limitations that need to be considered. First, although the PATH cohort is representative of the population from which it was recruited (Canberra and Queanbeyan, Australia [17]), it is not representative of the Australian population. Australian Bureau of Statistics (ABS) census data shows that the workforce in Canberra/Queanbeyan has almost 50% more professionals than the overall Australian population [37]. However, our previous research using the PATH cohort showed that workplace bullying was most common among professional and semi-professional occupations [21], and this is consistent with statistics in employment compensation data [38]. There is no reason to assume that the association between workplace bullying and suicidal ideation would be different in a nationally representative population. A further limitation is that the measures of workplace bullying and suicidal ideation were self-reported, and thus may not be as objective as other forms of assessment such as clinical, observational or administrative records (although these too have important limitations such as under-reporting in administrative records). Here, it is worth noting that the vast majority of prior research assessing workplace bullying has used self-report measures, with only single-item assessments of suicidality [12,13]. We also acknowledge that the small numbers in the suicidal ideation groups, and the small numbers exposed to verbal and physical threats of bullying, limited statistical power. However, this is the nature of these rare events, and thus we have taken a balanced view in interpreting both effect sizes and statistical significance. Finally, data on workplace bullying was only available at one time point. This restricted our ability to consider temporal and causal associations, and more robustly align past experiences of workplace bullying with prior experiences of psychosocial job adversity, depression and suicidality. While we excluded those with prior suicidal ideation in our sensitivity analyses to help reduce bias (and health selection), there is still the possibility that those with current suicidal ideation were more likely to report previous experiences of workplace bullying, artificially inflating the findings. 

## 5. Conclusions

In Australia, an average of eight people die by suicide and a further 30 people attempt suicide every day [39]. This study connects the existing literature demonstrating the adverse mental health consequences of workplace bullying with a need for more research identifying new risk factors for suicidality. We find that current and prior experiences of workplace bullying increase the odds of active suicidal ideation; but note that this, in part, reflects the broader adverse nature of the workplaces in which bullying takes place. Strengths of the study include the large community-based sample, the robust measure of active suicidal ideation, the inclusion of both self-labelling and behaviour-based measures of workplace bullying, and sensitivity analyses that restrict the sample to those with recent experience of suicidal ideation. In addition, the analyses consider the role of workplace bullying in connection with other psychosocial job adversities. Future prospective research should seek to track workplace bullying in association with suicidal ideation, job quality, job turnover and neuroticism over multiple time points to better understand the development and maintenance of suicidal ideation.

## Figures and Tables

**Table 1:** Socio-demographic characteristics of respondents all at wave 4 (N = 1448).

Characteristics	All Respondents	Non-Suicidal Respondents n = 1324 (91.4%)	Suicidal Respondents n = 122 (8.4%)	Chi Square Test/ANOVA (Non-SUICIDAL vs. suicidal)
	n (%) or Mean (SD)	n (%)/Mean (SD)	n (%)/Mean (SD)	*p*-Value
Sex				0.834
Male	699 (48.3)	638 (48.2)	60 (49.2)	
Female	749 (51.7)	686 (51.8)	62 (50.8)	
Age (years at time of interview)	55.0 (1.49)	54.97 (1.49)	54.89 (1.48)	0.555
Partner				0.053
Yes	1196 (82.6)	1104 (83.4)	91 (74.6)	
No	248 (17.1)	219 (16.5)	28 (23.0)	
Missing	4 (0.3)	1 (0.1)	3 (2.5)	
Education (years)	15.02 (2.19)	15.03 (2.18)	14.95 (2.24)	0.701
Weekly household income				**0.020**
<$1075	186 (12.9)	160 (12.1)	25 (20.5)	
<$1700	268 (18.5)	238 (18.0)	29 (23.8)	
<$2400	311 (21.5)	291 (22.0)	20 (16.4)	
$2400+	623 (43.0)	580 (43.8)	43 (35.3)	
Missing/not reported	60 (4.1)	55 (4.2)	5 (4.1)	
Employment status				0.715
Full time	1134 (78.3)	1039 (78.5)	94 (77.1)	
Part time	314 (21.7)	285 (21.5)	28 (23.0)	
Employment sector				0.087
Public sector (Commonwealth)	528 (36.5)	474 (35.8)	52 (42.6)	
Public sector (State/Territory)	226 (15.6)	205 (15.5)	21 (17.2)	
Private sector	475 (32.8)	435 (32.9)	40 (32.8)	
Not for profit/other	214 (14.8)	205 (15.5)	9 (7.4)	
Missing	5 (0.4)	5 (0.4)	0	
Occupational skill level				0.161
Professional	821 (56.7)	760 (57.4)	60 (49.2)	
Semi-professional	263 (18.2)	232 (17.5)	31 (25.4)	
Trade/manual	235 (16.2)	214 (16.2)	20 (16.4)	
Other	128 (8.8)	117 (8.8)	11 (9.0)	
Missing	1 (0.1)	1 (0.1)	0	
Long working hours				0.496
No (≤ 50)	1236 (85.4)	1127 (85.1)	107 (87.7)	
Yes (>50)	207 (14.3)	192 (14.5)	15 (12.3)	
Missing	5 (0.3)	5 (0.4)	0	
Number of chronic physical conditions				0.067
0	640 (44.2)	594 (44.9)	46 (37.7)	
1	548 (37.9)	501 (37.8)	45 (36.9)	
2 or more	260 (18.0)	229 (17.3)	31 (25.4)	
Job control				**0.004**
High	941 (65.0)	877 (66.2)	64 (52.5)	
Low	504 (34.8)	446 (33.7)	56 (45.9)	
Missing	3 (0.2)	1 (0.1)	2 (1.6)	
Job demands				0.267
Low	1078 (74.5)	991 (74.9)	85 (69.7)	
High	369 (25.5)	333 (25.2)	36 (29.5)	
Missing	1 (0.1)	0	1 (0.8)	
Job security				**<0.001**
High	1045 (72.2)	978 (73.9)	65 (53.3)	
Low	402 (27.8)	345 (26.1)	57 (46.7)	
Missing	1 (0.1)	1 (0.1)	0	
Workplace bullying				**0.005**
Never bullied	710 (49.0)	669 (50.5)	41 (33.6)	
Currently bullied	101 (7.0)	87 (6.6)	14 (11.5)	
Previously in current workplace	240 (16.6)	213 (16.1)	26 (21.3)	
Previously in previous workplace	330 (22.8)	293 (22.1)	36 (29.5)	
Cannot say	67 (4.6)	62 (4.7)	5 (4.1)	
Person-related bullying	1.29 (0.52)	1.27 (0.50)	1.47 (0.61)	**<0.001**
Work-related bullying	1.44 (0.68)	1.43 (0.67)	1.56 (0.72)	**0.033**
Violence-related bullying	1.03 (0.19)	1.03 (0.19)	1.06 (0.24)	0.229

Notes: Bold text indicates *p* ≤ 0.001.

**Table 2:** Odds (95% CI) of suicidal ideation associated with the ‘self-labelling’ measure of workplace bullying.

Characteristics	Model 1 ^a^ (Unadjusted)	Model 2 ^b^ (With Socio-Demographic and Health Covariates)	Model 3 ^c^ (With Socio-Demographic, Health, and Current and Prior Work Covariates)	Model 4 ^d^ (Excluding Those with Prior Suicidal Ideation)
Workplace bullying				
Never bullied (ref.)	1.00	1.00	1.00	1.00
Currently bullied	**2.63 (1.38–5.01)**	**2.37 (1.16–4.85)**	1.98 (0.93–4.23)	1.55 (0.52–4.63)
Previously in current workplace	**1.99 (1.19–3.33)**	**2.06 (1.19–3.57)**	**1.91 (1.07–3.38)**	1.74 (0.81–3.71)
Previously in previous workplace	**2.00 (1.26–3.20)**	**2.11 (1.28–3.48)**	**2.06 (1.21–3.5)**	**2.29 (1.15–4.54)**
Cannot say	1.32 (0.50–3.45)	1.15 (0.39–3.38)	0.88 (0.25–3.03)	1.02 (0.23–4.62)
Sex				
Male (ref.)		1.00	1.00	1.00
Female		0.72 (0.47–1.10)	0.67 (0.43–1.04)	0.93 (0.51–1.67)
Age (years)		0.94 (0.83–1.08)	0.92 (0.8–1.06)	0.94 (0.78–1.12)
Education (years)		1.03 (0.92–1.14)	1.01 (0.9–1.14)	0.96 (0.83–1.11)
Partner				
Yes		0.91 (0.54–1.53)	0.92 (0.53–1.59)	0.91 (0.43–1.91)
No (ref.)		1.00	1.00	1.00
Weekly household income				
<$1075		**2.23 (1.13–4.39)**	2.04 (0.98–4.25)	1.28 (0.46–3.55)
<$1700		1.68 (0.94–3.01)	1.73 (0.94–3.18)	1.44 (0.64–3.21)
<$2400		0.90 (0.49–1.62)	0.83 (0.45–1.55)	0.83 (0.38–1.83)
$2400+ (ref.)		1.00	1.00	1.00
Missing/not reported		1.51 (0.55–4.20)	0.82 (0.23–2.93)	0.35 (0.04–2.80)
Employment status				
Full time (ref.)		1.00	1.00	1.00
Part time		1.13 (0.68–1.90)	1.02 (0.59–1.77)	1.26 (0.62–2.56)
Employment sector				
Public sector (Commonwealth) (ref.)		1.00	1.00	1.00
Public sector (State/Territory)		0.81 (0.45–1.46)	0.79 (0.43–1.47)	0.52 (0.22–1.21)
Private sector		0.86 (0.52–1.42)	0.81 (0.47–1.37)	0.60 (0.30–1.21)
Not for profit/other		**0.39 (0.18–0.85)**	**0.35 (0.15–0.78)**	**0.30 (0.10–0.84)**
Occupational skill level				
Professional (ref.)		1.00	1.00	1.00
Semi-professional		1.58 (0.92–2.73)	1.53 (0.87–2.72)	0.59 (0.25–1.39)
Trade/manual		1.09 (0.56–2.13)	0.77 (0.36–1.64)	0.54 (0.20–1.46)
Other		1.13 (0.52–2.46)	1.07 (0.46–2.49)	0.88 (0.29–2.67)
Long working hours				
No (ref.)		1.00	1.00	1.00
Yes		0.83 (0.44–1.56)	0.85 (0.44–1.67)	0.90 (0.38–2.12)
Number of chronic physical conditions				
0 (ref.)		1.00	1.00	1.00
1		1.14 (0.72–1.79)	1.18 (0.73–1.89)	0.79 (0.43–1.46)
2 or more		1.58 (0.94–2.66)	1.66 (0.96–2.85)	1.10 (0.54–2.25)
Current job control				
High (ref.)			1.00	1.00
Low			1.07 (0.66–1.74)	1.20 (0.64–2.25)
Current job demands				
Low (ref.)			1.00	1.00
High			1.2 (0.71–2.01)	1.31 (0.66–2.58)
Current job security				
High (ref.)			1.00	1.00
Low			**2.28 (1.46–3.55)**	**2.51 (1.40–4.50)**
Prior job control				
High (ref.)			1.00	1.00
Low			**1.65 (1.02–2.67)**	**2.31 (1.25–4.29)**
Prior job demands				
Low (ref.)			1.00	1.00
High			0.96 (0.59–1.58)	0.93 (0.49–1.77)
Prior job security				
High (ref.)			1.00	1.00
Low			0.86 (0.52–1.44)	0.80 (0.40–1.58)

Notes: ^a^ Unadjusted model (n = 1446). ^b^ Model adjusted for sex, age, partner status, years of education, household income, employment mode, employment sector, occupational skill level, long working hours, and chronic physical conditions (n = 1410). ^c^ Model adjusted for the covariates in model 2 and current and prior job covariates (n = 1372). ^d^ Model adjusted for the covariates in model 3 and excluding those who had suicidal ideation in the previous wave (n = 1271). Bold text indicates *p* ≤ 0.001.

## Supplementary Materials

The following are available online at https://www.mdpi.com/1660-4601/17/4/1448/s1, Table S1: Odds (95% CI) of suicidal active and passive ideation associated with the ‘self-labelling’ measure of workplace bullying.
