# Supplementary material for: Workplace Bullying and Suicidal Ideation: Findings from an Australian Longitudinal Cohort Study of Mid-Aged Workers
Source: Int J Environ Res Public Health. 2020 Feb 24;17(4):1448. doi: 10.3390/ijerph17041448 (PMC7068571; doi:10.3390/ijerph17041448)
Supplement: Supplementary file 1 [file ijerph-17-01448-s001.pdf]

Supplementary Material

Table S1. Odds (95% CI) of suicidal active and passive ideation associated with the 'self-labelling' measure of workplace bullying.

	**Model 1[a] (unadjusted)**	**Model 2[b] (with socio-demographic and health covariates)**	**Model 3[c] (with socio-demographic, health, and current and prior work covariates)**	**Model 4[d] (excluding those with prior suicidal ideation)**
Workplace bullying				
Never bullied (ref.)	1.00	1.00	1.00	1.00
Currently bullied	**2.37 (1.37–4.11)**	**2.15 (1.19–3.89)**	**1.89 (1.01–3.52)**	1.09 (0.42–2.84)
Previously in current workplace	**1.81 (1.18–2.77)**	**1.79 (1.14–2.81)**	**1.66 (1.04–2.65)**	1.31 (0.70–2.46)
Previously in previous workplace	**2.27 (1.57–3.30)**	**2.30 (1.55–3.42)**	**2.23 (1.47–3.37)**	**2.52 (1.50–4.24)**
Cannot say	0.94 (0.39–2.27)	0.83 (0.32–2.15)	0.66 (0.23–1.93)	0.89 (0.26–3.04)
Sex				
Male (ref.)		1.00	1.00	1.00
Female		0.92 (0.65–1.29)	0.91 (0.64–1.31)	1.28 (0.80–2.06)
Age (years)		0.95 (0.85–1.07)	0.94 (0.84–1.06)	0.93 (0.80–1.07)
Education (years)		1.07 (0.98–1.17)	1.06 (0.97–1.17)	1.05 (0.93–1.18)
Partner				
Yes		0.83 (0.55–1.27)	0.84 (0.54–1.30)	0.79 (0.44–1.42)
No (ref.)		1.00	1.00	1.00
Weekly household income				
<$1,075		**1.82 (1.04–3.18)**	1.64 (0.90–2.97)	1.24 (0.56–2.74)
<$1,700		1.52 (0.95–2.44)	1.53 (0.94–2.5)	1.46 (0.77–2.74)
<$2,400		0. 87 (0.55–1.40)	0.86 (0.53–1.39)	0.67 (0.35–1.30)
$2,400+ (ref.)		1.00	1.00	1.00
Missing/not reported		1.34 (0.58–3.06)	0.97 (0.38–2.47)	0.70 (0.20–2.45)
Employment status				

Full-time (ref.)	1.00	1.00	1.00
Part-time	1.08 (0.71–1.64)	1.00 (0.65–1.56)	1.04 (0.59–1.85)
Employment sector			
Public sector (Commonwealth) (ref.)	1.00	1.00	1.00
Public sector (State/Territory)	0.82 (0.51–1.33)	0.79 (0.48–1.30)	0.59 (0.30–1.19)
Private sector	0.81 (0.53–1.22)	0.75 (0.49–1.16)	0.71 (0.40–1.24)
Not for profit/other	0.62 (0.36–1.06)	0.59 (0.34–1.02)	0.58 (0.29–1.17)
Occupational skill level			
Professional (ref.)	1.00	1.00	1.00
Semi-professional	1.49 (0.95–2.35)	1.50 (0.94–2.42)	1.05 (0.55–2.01)
Trade/manual	1.58 (0.93–2.68)	1.31 (0.74–2.34)	1.13 (0.53–2.42)
Other	1.25 (0.67–2.34)	1.28 (0.66–2.49)	1.29 (0.56–2.98)
Long working hours			
No (ref.)	1.00	1.00	1.00
Yes	1.00 (0.61–1.62)	1.02 (0.61–1.71)	1.31 (0.70–2.44)
Number of chronic physical conditions			
0 (ref.)	1.00	1.00	1.00
1	1.10 (0.77–1.58)	1.16 (0.80–1.69)	0.78 (0.48–1.29)
2 or more	1.43 (0.93–2.19)	1.50 (0.96–2.33)	1.28 (0.73–2.24)
Current job control			
High (ref.)		1.00	1.00
Low		1.02 (0.69–1.52)	1.09 (0.66–1.82)
Current job demands			
Low (ref.)		1.00	1.00
High		1.15 (0.76–1.75)	0.95 (0.54–1.65)
Current job security			
High (ref.)		1.00	1.00
Low		**1.89 (1.31–2.72)**	**1.80 (1.12–2.90)**
Prior job control			

High (ref.)	1.00	1.00
Low	1.48 (1.00–2.19)	**1.69 (1.01–2.81)**
Prior job demands		
Low (ref.)	1.00	1.00
High	1.10 (0.75–1.63)	1.24 (0.75–2.05)
Prior job security		
High (ref.)	1.00	1.00
Low	1.00 (0.66–1.51)	0.85 (0.49–1.49)

Notes: [a] Unadjusted model (n=1447). [b] Model adjusted for sex, age, partner status, years of education, household income, employment mode, employment sector, occupational skill level, long working hours, and chronic physical conditions (n=1411). [c] Model adjusted for the covariates in model 2 and low job control, high job demands, and high job insecurity (n=1373). [d] Model adjusted for the covariates in model 3 and excluding those who had suicidal ideation in the previous wave (n=1224).
